# The Polarization of the Coupling Strength of Interdependent Networks Stimulates Cooperation

**DOI:** 10.3390/e24050694

**Published:** 2022-05-14

**Authors:** Jinzhuo Liu, Yunchen Peng, Peican Zhu, Yong Yu

**Affiliations:** 1 School of Software, Yunnan University, Kunming 650504, China; jinzhuo.liu@hotmail.com (J.L.); 12019202388@mail.ynu.edu.cn (Y.P.); 2 School of Artificial Intelligence, Optics and Electronics (iOPEN), Northwestern Polytechnical University, Xi’an 710072, China; ericcan@nwpu.edu.cn

**Keywords:** evolutionary game theory, cooperation, interdependent network, coevolution

## Abstract

We introduce a mixed network coupling mechanism and study its effects on how cooperation evolves in interdependent networks. This mechanism allows some players (conservative-driven) to establish a fixed-strength coupling, while other players (radical-driven) adjust their coupling strength through the evolution of strategy. By means of numerical simulation, a hump-like relationship between the level of cooperation and conservative participant density is revealed. Interestingly, interspecies interactions stimulate polarization of the coupling strength of radical-driven players, promoting cooperation between two types of players. We thus demonstrate that a simple mixed network coupling mechanism substantially expands the scope of cooperation among structured populations.

## 1. Introduction

The complexity and scale of biology and human society largely depend on cooperation. Furthermore, the emergence and stability of cooperation is perplexing in light of Darwin’s notion of “survival of the fittest”; therefore, how to promote and consolidate cooperation has become a challenge in biology, sociology, psychology, and many other disciplines [1,2]. Evolutionary game theory [3,4,5,6,7,8] is an effective mathematical theoretical framework for studying the emergence and stability of cooperation under social dilemmas. At present, various mechanisms for promoting cooperation, such as kin selection [9], direct reciprocity [10], indirect reciprocity [11], voluntary participation [12,13], and group selection [14], have been identified.

One of the principal directions of the research on cooperation comes from the integration of game theory and network science [15,16,17]. Since Nowak and May [11] proposed the theory of network reciprocity, many studies have focused on the structure of interaction between individuals under different topologies, revealing the importance of spatial structure to cooperative evolution. These include small-world [18,19], scale-free networks [20,21] and adaptive networks [22,23,24]. Among them, interdependent networks [25,26,27,28,29,30,31], in which seemingly unrelated changes may lead to disastrous and unexpected consequences in another network, are widely used to study the evolution of cooperation [32,33,34,35,36].

Szolnoki [37] found that players on two different networks sharing information about strategy choices would enhance the evolution of cooperation. Especially in the coupling mode between interdependent networks, the most commonly used approach is to calculate the utility by combining the payoffs of participants in different networks [25,31]. Most methods assume that players are uniform, that is, they all use the same method or attitude to modify their current states. However, these studies ignore a simple but potentially fundamental mechanism—players’ different decision-making methods. The type of coupling between networks in the real world is often uneven, and our study committed to exploring the evolution of cooperation in interdependent networks by different coupling mechanisms. On this basis, we investigate the behavior of a heterogeneous population composed of conservative-driven players and radical-driven players. Conservative-driven players maintain a stable coupling strength in a way that minimizes individual risk and avoids overly risky personal choices, whereas radical-driven players break the stereotype and take risks for high rewards. In contrast to the monotonous growth of cooperation demonstrated in previous studies, the model shows a hump-like relationship between the level of cooperation and conservative participant density. In addition, in some cases, these heterogeneous populations do not have appropriate competition, but they form a strategic alliance to obtain better evolutionary outcomes. Further analysis shows that polarization of the coupling strength enhances cooperation.

The rest of this paper is organized as follows. We first describe the coevolution rule between strategy and coupling strength; then, we present the results. Finally, we summarize the main conclusions.

## 2. Methods

We consider a prisoner’s dilemma game on an interdependent network with two L×L square lattices with periodic boundary conditions. Initially, each player on both networks is either a cooperator (C) or defector (D) with equal probability. Then, players obtain payoffs based on pairwise interactions in their von Neumann neighborhood on the same layer via the following rules: mutual cooperation yields a reward R, while mutual defection leads to a punishment P. Under the mixed case, the cooperator obtains the sucker’s payoff S, and the defector obtains the temptation T. For simplicity, we consider a weak prisoner’s dilemma game, Refs. [38,39,40,41,42,43] where the payoff parameters are set as follows: R = 1, S = 0, T = *b*, P = 0. The *b* value represents the strength of the social dilemma.

Owing to the interdependence between the two networks, the fitness $U_{x}$ calculation of player *x* should take not only its own payoff $P_{x}$ into account but also the payoff $P_{x^{\prime }}$ of the corresponding player $x^{\prime }$ from the other network, the fitness $U_{x^{\prime }}$ of player $x^{\prime }$ on another network is calculated likewise, and the fitness $U_{x}$ and $U_{x^{\prime }}$ are calculated as follows

(1) $$\left\{\begin{array}{c} U_{x}=P_{x}+w_{x}P_{x^{\prime }}\\ U_{x^{\prime }}=P_{x^{\prime }}+w_{x^{\prime }}P_{x} \end{array}\right.$$

where $0\leq w_{x}\leq 1$ is a parameter to indicate the strength of the coupling between the two interdependent networks; the coupling strength is an intrinsic property of the player rather than the network.

In our setup, two types of players populate the environment. Conservative-driven players (CPs) avoid overly risky personal choices, and the coupling strength is constant (equal to 0.5). For radical-driven players (RPs), the evolution of the coupling strength $w_{x}$ follows the rule of teaching activity [44,45]. Specifically, if the RP spreads its strategy to any neighbor, $w_{x}$ will increase according to $w_{x}=w_{x}+\Delta$. In contrast, if the RP strategy fails to spread, $w_{x}$ will decrease according to $w_{x}=w_{x}-\Delta$, where the scaling factor Δ=0.1. To avoid frozen states, all RP coupling strengths are kept between $\left[w_{\min},w_{\max}\right]$ at all times, where $w_{\min}=0.01$ and $w_{\max}=1$.

We designate a population density μ as being conservative-driven. Subsequently, player *x* randomly selects one of their neighbors *y* on the same network and spreads their strategy to player *y* based on the imitation dynamics:

(2) $$W\left(s_{x}\to s_{y}\right)\;=\;\frac{1}{1+exp\left[\left[U_{y}-U_{x}\right]/K\right]}$$

where *K* quantifies the uncertainty related to the strategy adoption process; without loss of generality, we use K = 0.1 [46,47,48,49,50].

The evolutionary process is presented in Algorithm 1. The system uses Monte Carlo simulation to iterate forward and uses the asynchronous update rule, each player on the interdependent networks has a chance to update its strategy once on average during a full Monte Carlo step. To ensure that the system reaches a stable state, the maximum number of iterations t is equal to 100,000, and the system size varies from L = 100 to 400. All the data are averaged over up to 20 interdependent runs for each set of parameter values to ensure suitable accuracy. (These choices of simulation parameters allow to avoid finite size effects and to obtain accurate results.)
**Algorithm 1** Population game model1:Initialize the interdependent networks (*N*,$N^{\prime }$) of size=L×L, each player selects a cooperation or defection strategy with the same probability. Assign population label (RPs, CPs) for players, then assign coupling strengths $w_{x}$ based on the population label2:**for** s=0→MC_Steps **do**3:   **for** i=0→L*L **do**4:     select network N5:     x=random(L×L)6:     y=random (x’s neighbor)7:     calculate fitness of x and y according to Equation (1)8:     x passes strategy to y according to Equation (2)9:     **if** x∈RPs **then**10:        x adjusts the coupling strength $w_{x}$11:     **end if**12:     select network $N^{\prime }$13:     repeat steps 5-11 on another network $N^{\prime }$14:   **end for**15:**end for**

## 3. Results

**Figure 1:**
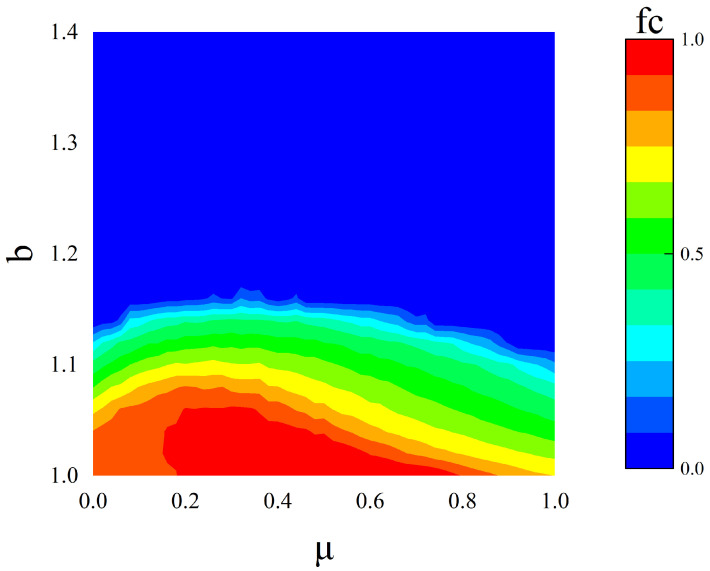
Frequency of cooperation ρc (color-coded according to the bar on the right). The phase diagram of the two-dimensional variable contains two parameters, the temptation to defect *b* and the ratio of conservative-driven players μ; the linear size of the lattices is L=400. All results are obtained for K = 0.1.

To obtain an overall profile, Figure 1 encodes the fraction of cooperators ρc depending on the temptation to defect *b* and the density of conservative-driven players within the population μ. The dependence of ρc on μ is non-monotonic, and there is a hump-shaped relationship between cooperation and the density of conservative-driven players, especially for small values of *b*. In structured populations, the combination of conservative and radical populations supports a higher level of cooperation than the pure population does, and cooperation peaks when the conservative-driven participant density is moderate. A further introduction of CPs makes cooperation a dominant behavior, but only while their density remains moderate. Specifically, cooperation peaks at a density of CPs of approximately 30% in the total population and gradually declines thereafter but without ever returning to zero (for b≤1.15). By comparison, when conservatives are absent, at μ=0, the cooperation frequency in pure populations of radical-driven players is well above the cooperation frequency in pure conservatives.

**Figure 2:**
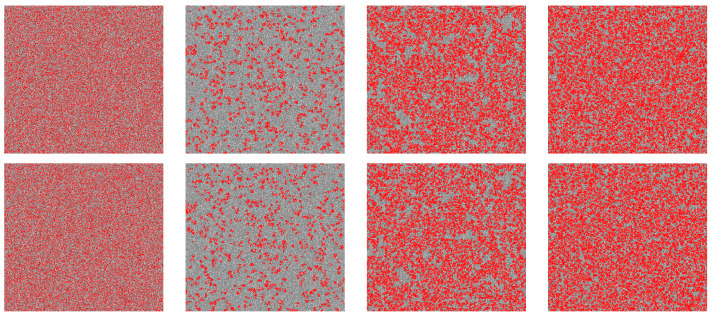
Typical snapshots of the evolution of strategies among different populations of players. Specifically, red (light red) denotes cooperation of radical-driven (conservative-driven) players, and grey (light grey) denotes defection of radical-driven (conservative-driven) players. From left to right, snapshots of the upper (top) and lower (bottom) networks after 0, 100, 1000, 10,000 MCS. The linear size of the lattices is L=400, and all results are obtained for b=1.1, K=0.1, and μ=0.3.

To further clarify how the heterogeneous population solves the social dilemma of coevolution based on interdependent networks, in Figure 2, we show a series of characteristic strategy distributions in which different colors are used to indicate not only to cooperators (red) and defectors (grey) but also to distinguish between conservative-driven (light) and radical-driven (dark) players. From a random initial state (ρc=50%), the system experiences a negative feedback process consisting of the enduring (END) period and the expanding (EXP) period [43,51]. In detail, cooperators are invaded by defectors and decrease quickly during the former period (ρc=20%), whereas the downward trend is stopped, and the remaining clusters of cooperators begin to expand in the EXP period. In the EXP period, the presence of conservative-driven and radical-driven players leads to significantly more unexpected consequences. The light-red cooperators are surrounded by dark-red cooperators on both networks (ρc=65%). This means that communities of conservative cooperators surrounded by radical cooperators play a vital role in promoting cooperation.

**Figure 3:**
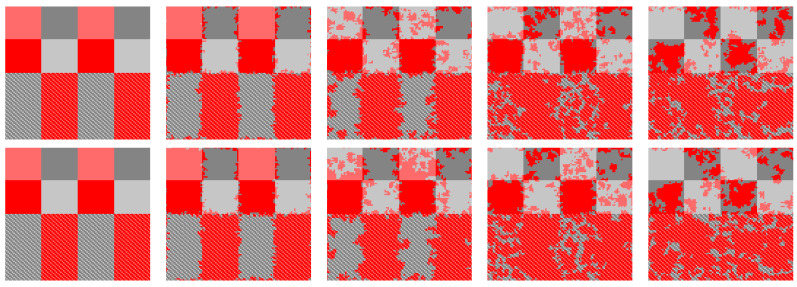
Evolution under certain initial conditions. The strategy of color matching of different populations is consistent with that in Figure 2. From left to right, the MC steps of the snapshots correspond to 0, 10, 100, 1000, and 10,000. The first (second) row of the snapshots corresponds to the strategy distribution of the top (bottom) network. All results are obtained for b=1.1, K=0.1. The linear size of the lattices is L=100.

To explore the phenomenon in Figure 2 more clearly, we launch the evolution from a prepared initial state. In Figure 3, the upper halves of each network are divided into eight square regions, each representing a pure population, and the lower halves are divided into four rectangular regions, each consisting alternately of two populations. Light-red cooperators in the upper halves are quickly occupied by defection, and cooperation is consolidated only in the clusters of dark-red cooperators. In contrast, light-red cooperators are more likely to be invaded by defectors than dark-red cooperators. By contrast, in the lower half, alternating between light red and dark red gradually eliminates the defectors and leads to a dominant position. In summary, once conservative-driven players are introduced into an environment surrounded by radical-driven players, they start to cooperate with each other. Conservative-driven players, by contrast, prefer to defect. Finally, the combination of conservative and radical participants tends to result in cooperation.

**Figure 4:**
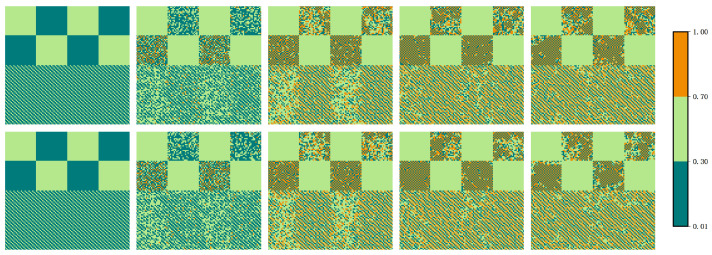
Typical snapshots of the evolution of the coupling strengths in Figure 3. From left to right, the MC steps of the snapshots correspond to 0, 10, 100, 1000, and 10,000. The first (second) row of snapshots corresponds to the distribution of the coupling strength of the top (bottom) network. The parameter values of this figure are the same as those in Figure 3.

Figure 4 further reports the coupling strength corresponding to Figure 3. For radical-driven players, the coupling strength of the cooperative clusters are dominated by the maximum value ($w_{\max}$) and the minimum value ($w_{\min}$). There is an equal proportion of $w_{\max}$ and $w_{\min}$ in the coupling strength of radical-driven players; $w_{\max}$ and $w_{\min}$ appear alternately on the lattice network. Thus, a phenomenon of self-organization similar to the alternate players in the lower halves of each network of Figure 3 emerges. For $w_{\max}-w_{\min}$ strategy pairs, for the cooperative pair based on the coupling strength of $w_{\max}$ and $w_{\min}$, RPs with coupling strength equal to $w_{\max}$ are responsible for spreading the strategy, and RPs with coupling strength equal to $w_{\min}$ learn the strategy. Under these circumstances, the strategies of CPs are more likely to be assimilated by the strategy of cooperative pairs.

**Figure 5:**
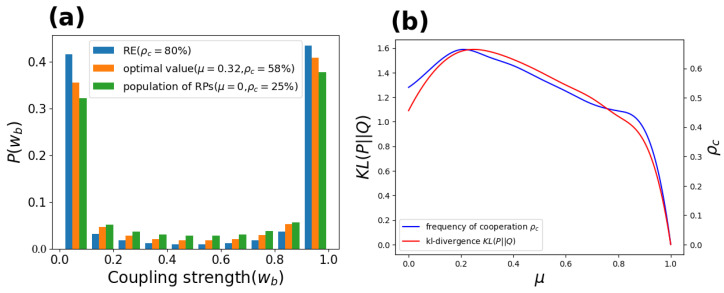
Panel (**a**): Coupling strength distribution of radical-driven cooperators. Polarization establishes the diversity of species and strengthens the cooperative behavior of the population. RE represents the coupling strength distribution of radical-driven players in the reconstructed environment in Figure 4. Panel (**b**): The portion of conservative-driven players μ corresponds to frequency of cooperation ρc and the value of KL divergence. The results are obtained for b=1.1, K=0.1, and L=400.

As discussed by Shi [31], the two-class society phenomenon is used, that is, the system is mainly occupied by players with large and low learning ability to help establish species diversity. To explore the impact of coupling strength on cooperative evolution, the distribution of coupling strength of radical-driven cooperators is reported in Figure 5a. Clearly, the coupling strength distribution shows a two-level trend, i.e., the cooperators with coupling strengths $w_{x}=1$ and $w_{x}=0.01$ account for the majority. Under the optimal value μ=0.3, this phenomenon is particularly obvious. By comparison, in our reconstructed environment (RE), the two-level differentiation phenomenon surpasses the other two situations. Furthermore, we use KL divergence to quantify the degree of polarization of the radical-driven population’s coupling strength (Figure 5b), which is $\mathrm{KL}\left(P||Q\right)=\sum P\left(w_{B}\right)\log\frac{P(w_{B})}{Q(w_{B})}$, $Q(w_{B})$ means uniform distribution. Notably, the curve of the frequency of cooperation is highly fitted to the curve of KL divergence, and both have peaks. Therefore, the promotion of cooperation is positively correlated with the polarization of coupling strength.

**Figure 6:**
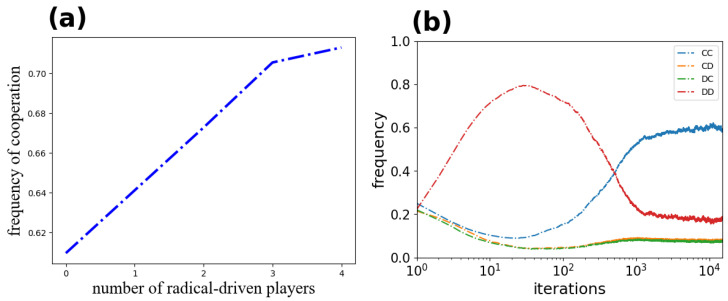
Panel (**a**): Frequency of cooperation of conservative-driven players with different numbers of radical-driven neighbors. Panel (**b**): Time courses of the frequency of CP–RP (population) strategy pairs. The results are obtained for b=1.1, K=0.1, L=400, and μ=0.3.

As elaborated above, communities composed of conservative players and radical players can effectively promote cooperation. In Figure 6a, the cooperation frequency of the conservative population increases with an increase in the number of participants in the surrounding radical population. In addition, we record changes in the frequency of strategy pairs formed by conservative players and radical players (Figure 6b): CC (concave) and DD (convex) interactions are the most common. Therefore, once conservative players are introduced into the environment, the two types of players soon start to cooperate with each other.

**Figure 7:**
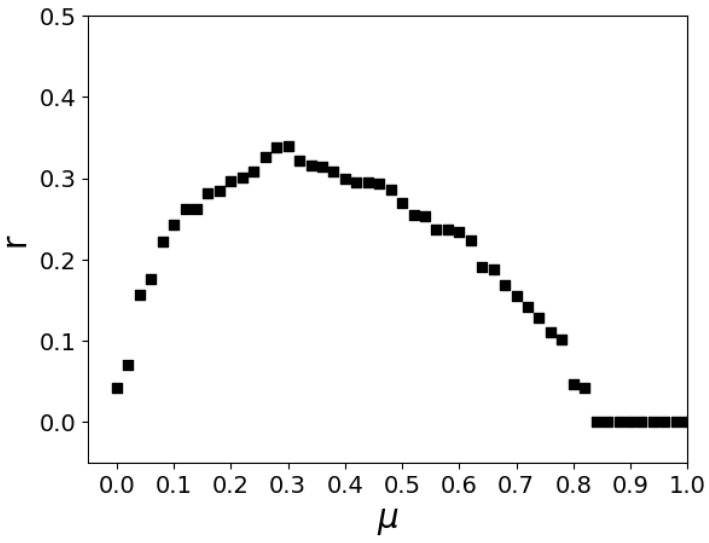
Relationship between r and μ in the equilibrium state. All the results are obtained for b=1.13 and K=0.1. Each data point results from an average of 20 independent runs.

To observe the coevolution of cooperation in interdependent networks, we define $r=\frac{N_{cc}}{L*L}$ as a measurement of the extent of the synchronization of evolution, where $N_{cc}$ denotes the number of interlayer C–C pairs between the two networks. We show *r* as a function of *μ* in Figure 7. When μ=0.3, *r* reaches its peak, which means that the evolution of the cooperation of the interdependent networks is synchronized; in fact, the synchronization of evolution is a direct consequence of interdependent network reciprocity, and the interaction between conservative-driven players and radical-driven players promotes such a consequence.

## 4. Conclusions

In summary, our study considered two populations with different decision-making mindsets. The agents based on these two thinking characteristics are mixed on the interdependent network. The two populations have different evolutionary dynamics, and there is an optimal ratio of CPs that can effectively promote cooperation. Specifically, CPs establish fixed coupling strength links with corresponding players on another layer, while the coupling strength of RPs evolves with the spread of the strategy. Because the ratio of the two populations affects the spatial structure, when the ratio of CPs is appropriate, the frequency of cooperation of the two populations reaches a peak. Then, we constructed a special spatial distribution consisting alternately of two populations. We find that RPs are the initiators of cooperation, and their coupling strength is polarized and consolidates their own cooperation. In contrast, CPs are more inclined to defect, but CPs learn the cooperative behavior from nearby RPs and inhibit the occurrence of defection. Moreover, interaction between the two populations jointly promotes cooperation.

Szolnoki [39] solve traditional social dilemma through network reciprocity, Perc and Wang [25,45] introduced the interlayer coupling mechanism of a multilayer network to promote cooperation, and our research through mixed network coupling mechanisms helps to resolve social dilemmas beyond traditional network reciprocity. This work on multilayer networks is motivated by the fact that networks of networks are often a significantly more apt description of real-life systems than isolated networks [52,53], rewarding evolutionary fitness by enabling links between populations. Directions for future research are many, for example, a multilayer network model can describe the process of disease transmission more intuitively, with disease transmission on one layer and epidemic prevention measures such as vaccination on another layer. Exploring the coupling mechanism between layers will provide a novel way for epidemiological research [54,55,56].

## Data Availability

Not applicable.
